# Infrapatellar fat pad adipose-derived stem cells co-cultured with articular chondrocytes from osteoarthritis patients exhibit increased chondrogenic gene expression

**DOI:** 10.1186/s12964-021-00815-x

**Published:** 2022-02-12

**Authors:** Christopher C. H. Mak, Kendrick To, Karim Fekir, Roger A. Brooks, Wasim S. Khan

**Affiliations:** 1 School of Clinical Medicine, Hills Road, Cambridge, CB2 0QQ UK; 2 Department of Pharmacology, University of Cambridge, Tennis Court Road, Cambridge, CB2 1PD UK; 3 Division of Trauma and Orthopaedic Surgery, Department of Surgery, Addenbrooke’s Hospital, University of Cambridge, Hills Road, Cambridge, CB2 0QQ UK; 4 Versus Arthritis Tissue Engineering and Regenerative Therapies Centre, Division of Trauma and Orthopaedic Surgery, Department of Surgery, Addenbrooke’s Hospital, University of Cambridge, Hills Road, Cambridge, CB2 0QQ UK

**Keywords:** Mesenchymal stromal cells, Co-culture, Chondrogenesis, Cartilage, Cell-based therapy, Regenerative medicine, Osteoarthritis

## Abstract

**Aim:**

The variable results in clinical trials of adipose tissue-derived stem cells (ASCs) for chondral defects may be due to the different ex vivo culture conditions of the ASCs which are implanted to treat the lesions. We sought to determine the optimal in vitro chondrocyte co-culture condition that promotes infrapatellar fat pad-derived (IFPD) ASC chondrogenic gene expression in a novel co-culture combination.

**Methods:**

In our study, we utilized an in vitro autologous co-culture of IFPD ASCs and articular chondrocytes derived from Kellgren–Lawrence Grade III/IV osteoarthritic human knee joints at ASC-to-chondrocyte seeding log ratios of 1:1, 10:1, and 100:1. Gene expression following in vitro co-culture was quantified by RT-qPCR with a panel comprising COL1A1, COL2A1, COL10A1, L-SOX5, SOX6, SOX9, ACAN, HSPG2, and COMP for chondrogenic gene expression.

**Results:**

The chondrogenic gene expression profiles from co-cultures were greater than would be expected from an expression profile modeled from chondrocyte and ASC-only monocultures. Additionally, chondrogenic gene expression decreased with increasing ASC-to-chondrocyte seeding ratios.

**Conclusions:**

These findings provide insight into the mechanisms underlying clinical ASC therapies and signifies that IFPD ASCs pre-conditioned by chondrocyte co-culture may have improved chondrogenic potential for cartilage repair. This model can help further understand IFPD ASCs in chondral and osteochondral repair and the chondrogenic pathways involved.

**Video Abstract**

**Supplementary Information:**

The online version contains supplementary material available at 10.1186/s12964-021-00815-x.

## Background

Osteoarthritis is characterized by degradation of articular cartilage, influenced by altered mechanical loading and exacerbated by localized inflammation [1]. This causes pain and disability in affected patients, and a significant disease burden globally. Articular cartilage has a limited ability to self-repair. While native joint-resident mesenchymal stromal cells (MSCs) mobilize and differentiate at sites of chondral lesions, their numbers are inadequate in vivo, thus spurring the development of intra-articular cell-based therapies using enriched or ex vivo expanded ASCs. With increasing reognition of the importance of early intervention in osteoarthritis [2], ASC therapies for early-stage osteoarthritis are under intense investigation [3–6]. Although these animal and clinical trials demonstrated pain reduction, the extent of cartilage repair has been variable [7, 8].

While bone marrow-derived MSCs and umbilical cord blood-derived MSCs have been investigated in clinical trials, they are limited by low relative abundance and ease of isolation respectively. Infrapatellar Fat Pad-Derived (IFPD) ASCs on the other hand exhibit a high relative abundance, ease of isolation, and high proliferation potential [9–11]. The fat pad is an intra-articular structure anatomically located near cartilage, and has been shown to be a source of ASCs capable of chondrogenic differentiation and the repair of osteochondral defects in vivo [12]. Its efficacy in treating human knee osteoarthritis through intra-articular injection has also been described in clinical trials [3]. The relative abundance avoids the need for expansion in vitro to increase cell numbers, and the associated dedifferentiation of MSCs [13–19]. Unpassaged cells that have not been expanded ex vivo more closely resemble cells in vivo and represent a more attractive option as a therapeutic intervention.

Our study investigates chondrogenic gene expression in ASCs with a novel in vitro autologous co-culture of early passage (p0) IFPD ASCs and chondrocytes from osteoarthritic knee joints using different ASC-to-chondrocyte seeding ratios. The primary objective of this study was to determine whether and to what extent the chondrogenic gene expression of ASCs is stimulated when co-cultured with chondrocytes. Our study aims to provide insight into the mechanisms underlying chondral repair with ASC therapies and demonstrate the potential presence of ASC-chondrocyte crosstalk in inducing chondrogenic gene expression in ASCs [20]. This information will allow us to better understand how these ASCs can be applied in cartilage repair and suggest directions for future clinical trials.

## Materials and methods

### Isolation of IFPD ASCs and chondrocytes

Human tissue containing articular cartilage and infrapatellar fat pad from total knee arthroplasties for osteoarthritis (IRAS: 247368, REC: 18/NV/0545, July 2018) was obtained following informed consent and handled in accordance with ethical guidelines outlined by the Human Tissue Act 2004 [21]. All patients had grade III or IV osteoarthritis of the knee according to the Kellgren and Lawrence classification [22]. After discarding tissue infected at source (n = 2), and tissue that had inadequate size (fat pad < 5 mL or Cartilage < 2 g; n = 1), 15 samples were cultured from both male (n = 3) and female (n = 12) patients ranged from 54 to 89 years of age, two further samples were discarded due to contamination with bacteria following culture expansion, and two samples did not reach 90% confluence after 21 days in culture. RNA extraction was performed on 13 samples, two were rejected due to low mRNA purity (260/280 ratio < 1.8) and two samples with low housekeeping gene expression (cycle threshold > 30) were discarded. Nine samples remained (aliquots of each used for proliferation assay), four samples were used in the pilot study and five samples contributed to the project data.

Infrapatellar fat pad samples were bathed in PBS and stored within a sterile container at 4 °C and were processed within 3 h of harvest. ASCs were extracted from the entire infrapatellar fat pad sample by mechanical dissection with a sterile scalpel and enzymatic digestion with 20 mL of sterile 0.2% Collagenase A (Sigma Aldrich) solution (passed through a 0.2 μm filter) made with 40 mg of Collagenase A into 20 mL of Dulbecco’s Modified Eagle Medium (DMEM, ThermoFisher), for 90 min at 37 °C with constant agitation. The digest was eluted through a 70 μm filter. An equal volume of basal medium composed of low-glucose Dulbecco’s Modified Eagle Medium (DMEM, ThermoFisher) supplemented by 10% Fetal Bovine Serum (FBS, ThermoFisher) and 1% penicillin/streptomycin (ThermoFisher) was added and the digest centrifuged at 300 g for 5 min. The supernatant was discarded, and the pellet resuspended in 10 mL of PBS. The resuspended cells underwent the ASC characterization steps described below.

### IFPD ASC characterization

The IFPD cells were expanded to 90% confluence or greater at passage 0 (p0), with media changes every two to three days and were then detached by addition of 2 mL of 1× TryplE Reagent (ThermoFisher), centrifuged and resuspended in 10 mL of 0.2% BSA-EDTA in PBS (ThermoFisher), and stained on ice with monoclonal antibody-fluorophores (Additional file 1: Table S1) for surface markers defined by the minimal criteria of stromal stem cells stipulated by the International Society for Cellular Therapy (ISCT) [23]. Aliquots of confluent culture-expanded p0 cells from two subjects were treated with FcR blocker (Miltenyi Biotec) and FACS buffer (Sigma Aldrich), then were analyzed by flow cytometry against a panel of markers (Additional file 1: Table S1) using the BD LSRFortessa™ Cell Analyzer.

### IFPD ASC trilineage differentiation

To determine their tri-lineage potential, aliquots of the cell suspension were cultured separately in chondrogenic (1:200 TGFβ1, 1:500 Vitamin C, 1:10,000 Dexamethasone in ITS & Proline supplemented basal medium,, ThermoFisher & Sigma Aldrich), osteogenic (1:10 BGP, 1:100 Vitamin C, 1:10,000 Dexamethasone in basal medium, ThermoFisher & Sigma Aldrich), and adipogenic medium (StemPro Adipogenesis Differentiation Kit). Chondrogenic differentiation was performed with a suspension of approximately 2 × 106 cells a culture in a test-tube that subsequently formed a 3D spheroid pellet in chondrogenic media (Additional file 1: Table S2). Cells for osteogenic and adipogenic differentiation were cultured as monolayers in 12-well plates in 2 mL of osteogenic and adipogenic media (Additional file 1: Table S3). Osteogenic differentiation required seeding of 2 × 104 cells/cm^2^ and adipogenic differentiation required seeding at 4 × 104 cells/cm^2^. Controls were cultured in basal medium in both 3D and monolayer. Differentiated cells were fixed and stained respectively by Alcian Blue, Alizarin Red S, and Oil Red O (Sigma Aldrich) for chondrogenic, osteogenic, and adipogenic differentiation. Chon-drogenic pellets were embedded in paraffin wax and sectioned using a microtome before staining.

Chondrocytes were extracted from the articular cartilage on the medial and lateral femoral condyles, tibial plateau, and patellar facets by mechanical and chemical digestion by 20 mL of sterile 0.2% Collagenase A solution for 20 h at 37 °C with constant agitation. The digest was eluted through a 70 μm filter to remove large non-cellular aggregate, centrifuged at 400*g* for 10 min and resuspended in 10 mL of PBS. The cells were counted using Trypan Blue cell viability assay on a hemocytometer [24].

### Seeding IFPD ASCs and chondrocytes in co-culture

The study involved co-culturing donor-matched p0 ASCs with p0 chondrocytes in monolayer directly in T25 flasks, each with 4 mL of basal media comprising of low-glucose Dulbecco’s Modified Eagle Medium (DMEM, ThermoFisher) supplemented by 10% Fetal Bovine Serum (FBS, ThermoFisher) and 1% penicillin/streptomycin (ThermoFisher) and the digest centrifuged at 300*g* for 5 min, at 37 °C. p0 ASCs and chondrocytes were plated into co-cultures immediately following digestion and resuspension. We conducted a pilot study comparing linear (n = 2) and logarithmic (n = 2) seeding ratios and opted for the latter as we were using p0 ASCs. p0 IFPD ASCs are a heterogeneous population comprising of the stromal vascular fraction including multipotent ASCs and more committed progenitors [25], up to 90% of the isolate can be formulated of other cell types prior to the first media change. Most non-ASC cells were removed during the isolation process by collagenase digestion, as discarded suspension during the first media change due to low plastic adherence [26], and ameliorated by the selective proliferation of plastic adherent ASCs during clonal expansion, as non-adherent cells are lost during media changes [27, 28]. Controlled at 5000 cells/cm^2^ final concentration, cells were seeded at increasing ASC-to-chondrocyte seeding log ratios at 1:1, 10:1, and 100:1, accompanied by positive and negative controls of chondrocyte-only and ASC-only monoculture respectively (n = 5).

### Cell proliferation

The Quant-iT Picogreen dsDNA assay (ThermoFisher) was performed to assess cell count and proliferation as per standard protocol [29], this was performed on four samples from the pilot study and five samples from the main study. Cells were detached by TryplE Reagent, centrifuged and resuspended with 1 mL of lysis buffer comprising 10 mM Tris, 1 mM EDTA, and 0.2% Triton X-100. Calibrated with nine patients, the spectrophotometer (Leica) measured absorbance at 520 nm which was directly proportional to the concentration of DNA, and thereby the number of cells per flask. This was supplemented by phase-contrast microscopy (Leica) documented by light micrographs delineating cell morphology and confluence. Cell proliferation was documented from seeding to harvest, with cell morphology delineated by phase-contrast microscopy. The cellular debris in the p0 ASC population and the non-ASC populations were re-moved at media change due to non-adherence to plastic.

Total DNA as a measure of cell number was quantified at harvest for each seeding ratio using the Picogreen dsDNA spectrophotometric assay by obtaining a calibration curve, which carries the advantage of low signal interference from proteins and single-strand nuclei acid. Culturing on 25 cm^2^ monolayers over a fixed time period of 18–21 days.

### Quantitation of mRNA and cDNA synthesis

Following coculture, cells were harvested at p0 at 90% confluence after 18–21 days of culture with QIAzol Lysis Reagent (Qiagen). RNA was extracted using the Direct-zol RNA MicroPrep Kit (Zymo Research) [30]. mRNA purity was assessed by its 260/280 ratio by quantitation at 260 nm absorbance with a NanoDrop spectrophotometer (ThermoFisher). cDNA was synthesized from RNA samples with a 260/280 nm absorbance ratio > 1.8 using the QuantiTect Reverse Tran-scription Kit and protocol [31].

### Quantitative real-time PCR

Quantitative real-time PCR (RT-qPCR) was performed by the addition of SYBR Green premix (ThermoFisher), corresponding 1 μM forward and 1 μM reverse primers, and 10 ng of cDNA per well to a final volume of 5 μl per well on a 96-well plate. The reaction was initiated with a 5-min pre-incubation at 95 °C, and 40 cycles of 10 s at 95 °C, 30 s at 60 °C for denaturation, annealing, and extension. A melt curve analysis was performed for 15 s at 95 °C, 60 s at 60 °C, and 15 s at 95 °C. Copy numbers per gene of interest were determined by cycle threshold (△Cτ) normalized against the housekeeping gene HPRT1. Individual samples or entire batches with ASC or Chondrocyte monocultures showing Cτ [HPRT1] > 30 were rejected. Primers for the chondrogenic RT-qPCR panel are shown in Table 1 [32–34]. Housekeeping HPRT1 expression was consistent across all intervals, with a mean Cycle Threshold (Cτ) of 24.90 ± 0.19. Expression cut-off was determined to be at Cτ 35 from Cτ values given by negative controls, corresponding to − ΔCτ of − 11. The cut-off was determined since further cycles would not give useful information due to the noise of amplified sparse DNA from the basal level of mRNA synthesis or surroundings [35].

**Table 1:** Primers designed with existing studies and PrimerBLAST, manufactured by Sigma Aldrich

Gene	Forward primer	Reverse primer
HPRT1	5′-TGACACTGGCAAAACAATGCA-3′	5′-GGTCCTTTTCACCAGCAAGCT-3′
COL1A1	5′-ATGCCTGGTGAACGTGGT-3′	5′-AGGAGAGCCATCAGCACCT-3′
COL2A1	5′-AACCAGATTGAGAGCATCCGC-3′	5′-CGATAACAGTCTTGCCCCACTTAC-3′
COL10A1	5′-CACCTTCTGCACTGCTCATC-3′	5′-GGCAGCATATTCTCAGATGGA-3′
L-SOX5	5′-GAATGTGATGGGACTGCTTATGTAGA-3′	5′-GCATTTATTTGTACAGGCCCTACAA-3′
SOX6	5′-CACCAGATATCGACAGAGTGGTCTT-3′	5′-CAGGGTTAAAGGCAAAGGGATAA-3′
SOX9	5′-GCAGGCGGAGGCAGAGGAG-3′	5′-GGAGGAGGAGTGTGGCGAGTC-3′
ACAN	5′-AGGGCGAGTGGAATGATGTT-3′	5′-GGTGGCTGTGCCCTTTTTAC-3′
HSPG2	5′-TCAGTCCCTTGTCACCATCCA-3′	5′-TAAGCTGCCTCCACGCTTAT-3′
COMP	5′-AACAGTGCCCAGGAGGAC-3′	5′-TTGTCTACCACCTTGTCTGC-3′

### Relative gene expression

Gene expression was determined by the 2−ΔΔCτ method [36]. − ΔCτ was used to demonstrate the different relative levels of expression of different genes. The positive control (chondrocyte) monoculture was used as reference during 2−ΔΔCτ calculations since it has a consistent phenotype as a terminally differentiated cell. The expected expression, as an indicator of chondrogenic differentiation was determined by calculating the ratio of ASC-to-chondrocyte gene expression observed from their respective control monocultures; the expected 1:1, 10:1 and 100:1 expression was derived from the fractional summation of the observed expression from 1/2, 10/11 and 100/101 of the positive control ASC monoculture respectively and 1/2, 1/11 and 1/101 of the negative control chondrocyte monoculture respectively.

### Statistical analysis

All experiments were repeated independently in triplicate. Statistical analysis was performed with GraphPad Prism 8. Depending on the homoscedasticity and normality of data determined by independent F-tests and Shapiro–Wilk tests respectively, Student’s t-test (homoscedastic), Welch’s t-test (heteroscedastic), or Mann–Whitney U test (skewed non-parametric distribution) was adopted in pairwise comparisons. One-way Fisher’s ANOVA with post hoc Tukey’s HSD (homoscedastic) or One-way Welch’s ANOVA with post hoc Dunnett’s test (heteroscedastic) was adopted for multiple comparisons.

## Results

### Characterization of ASCs

**Fig. 1:**
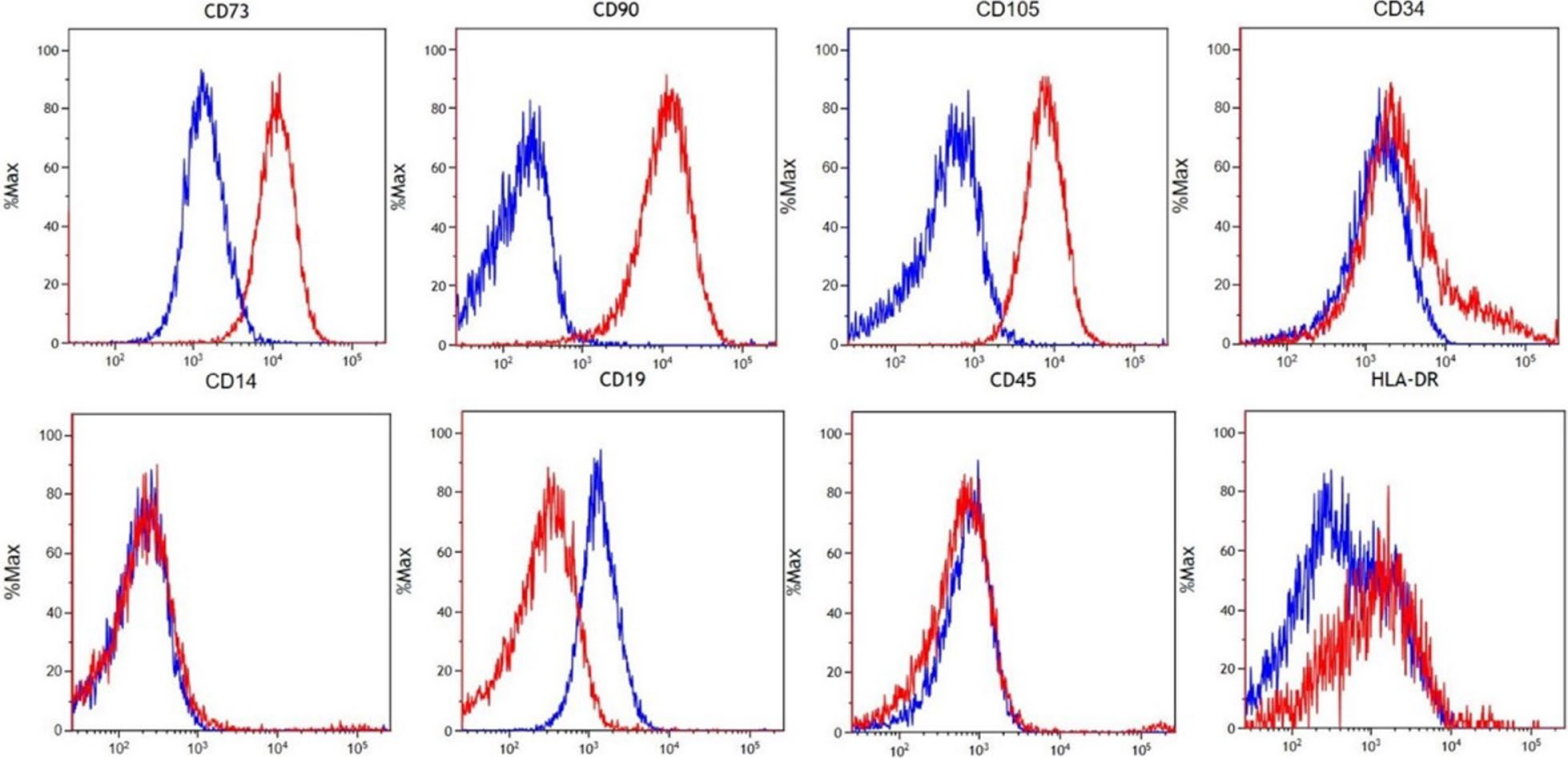
Epitope characterization of IFPD ASCs using the ISCT panel confirms expression of key markers. Top row markers are expressed: CD73, CD90, CD105, and a sub-population of CD34 (Stained (Red) readouts greater than negative (Blue)). Bottom row markers lack expression: CD14, CD19, CD45, and HLA-DR (stained HLA-DR population overlaps onto negative population)

**Fig. 2:**
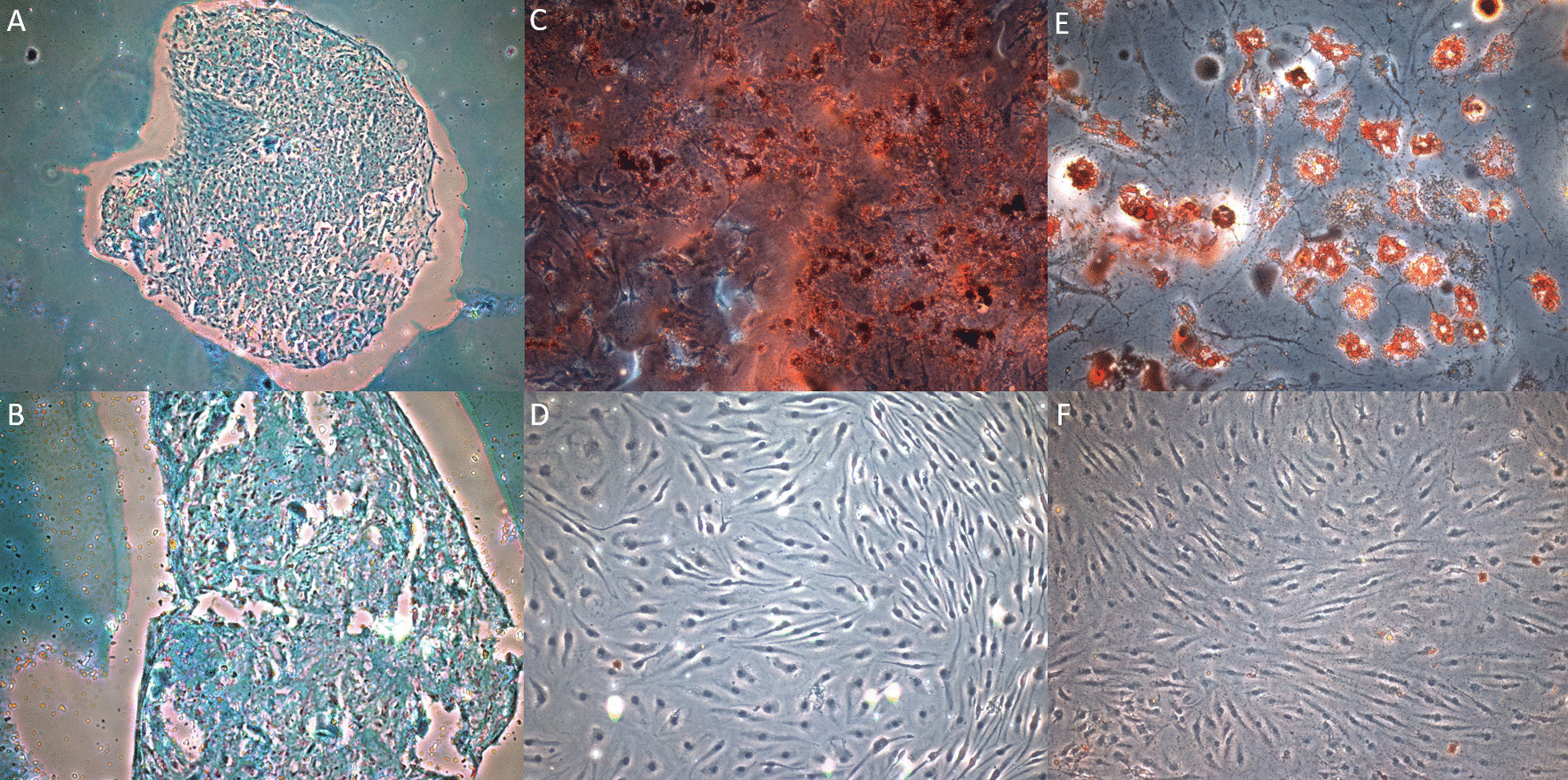
Histology demonstrating the trilineage potential of IFPD ASCs. **A** 40× and **B** 100× magnifications of an Alcian Blue stained chondrogenic pellet section indicating the presence of glycosaminoglycans found in cartilage ECM from ASCs cultured in chondrogenic medium. ASCs were unable to form a pellet in control using non-differentiating medium from the experiment protocol, hence no control staining is provided. **C** 100× magnification of Alizarin Red S stained calcium deposits demonstrating osteogenesis in ASCs cultured in osteogenic medium. **D** 200× magnification of Alizarin Red S applied to ASCs cultured in control medium, no calcium deposits demonstrated. **E** 100× magnification of Oil Red O stained lipid vesicles demonstrating adipogenesis in ASCs cultured in adipogenic medium. **F** 200× magnification of Oil Red O stained ASCs cultured in control medium, no lipids vesicles demonstrated

As per the ISCT, the ASCs were characterized and demonstrated:

- 1. Plastic adherence.
- 2. Expression of CD73, CD90 & CD105, and absent expression of CD14, CD19, CD45 & HLA-DR. The CD34 ex-pression was heterogeneous, with 14.07% of cells showing positive expression Additional file 1: Table S4. We found homogenous negative HLA-DR expression between the negative and stained populations and did not observe a clear separation on the single-parameter histogram (Fig. 1). Note that the ISCT criterion of absent CD34 for ASC is challenged as not entirely applicable to freshly isolated and minimally cultured ASCs [37, 38]
- 3. Chondrogenic, osteogenic, and adipogenic differentiation in vitro (Fig. 2).

### Proliferation of ASC and chondrocyte across seeding ratios

**Fig. 3:**
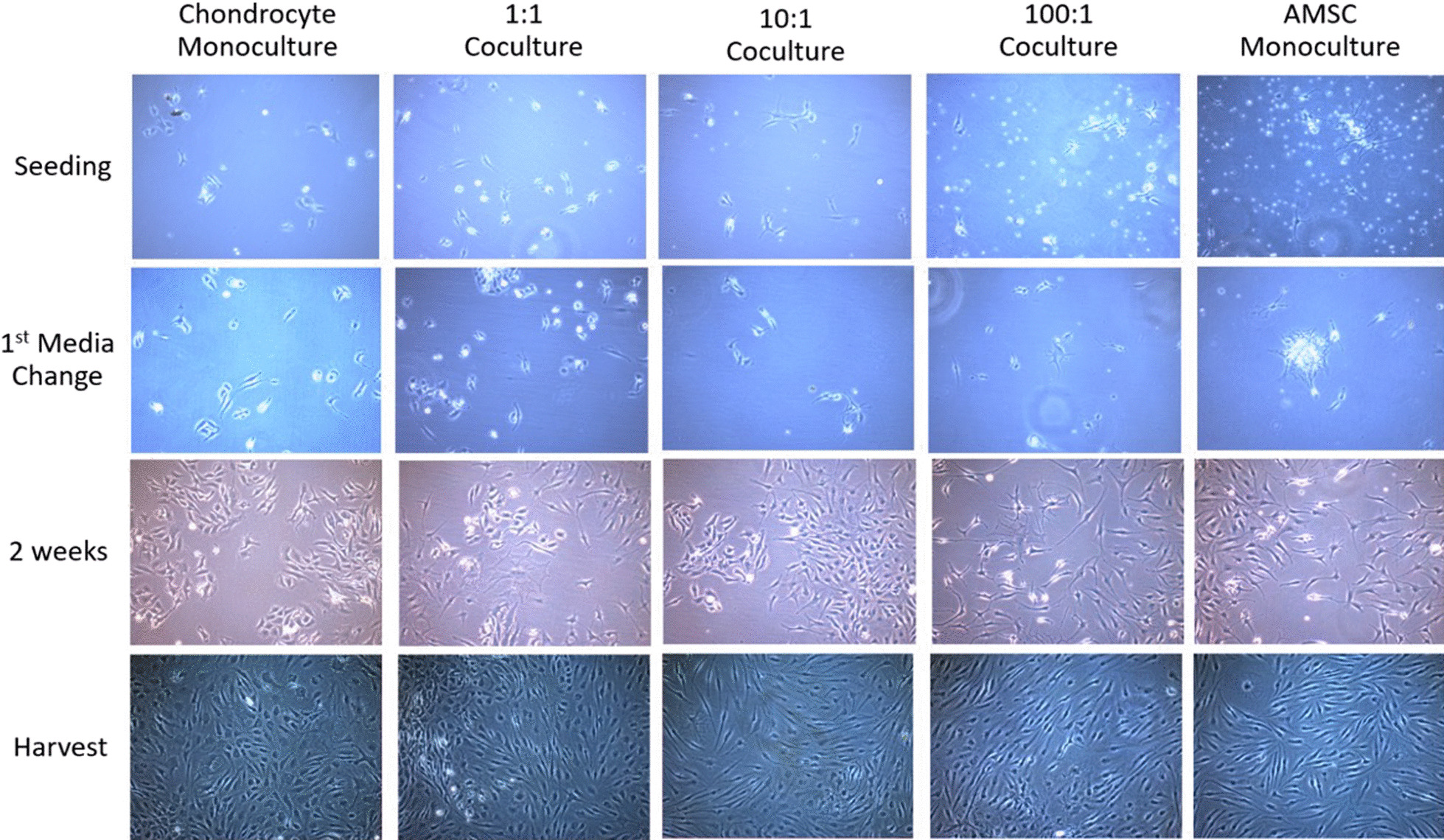
Photomicrograph of proliferating co-culture intervals and monoculture controls from seeding to harvest showing no difference in cell morphology at confluence. Black bar indicates a distance of 100 μm

Cell proliferation was documented from seeding to harvest, and Fig. 3 shows the cell morphology delineated by phase-contrast microscopy.

**Fig. 4:**
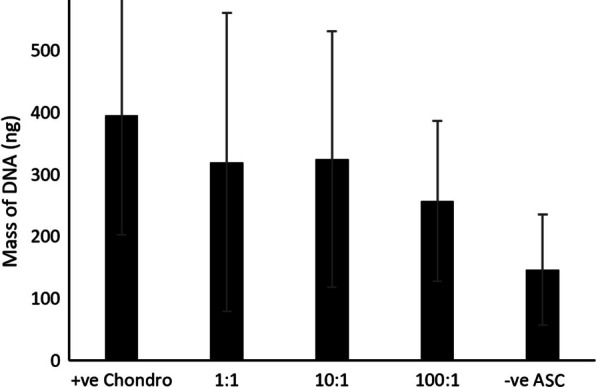
Quantity of DNA (mean ± SD) across different seeding ratios were not significantly different; positive chondrocyte control (+ ve Chondro), negative ASC control (-ve ASC), used in pilot and experimental trials (n = 9)

The mean volume of IFP per patient knee was 7.9 mL (± 8.0 mL SD, 5.0–37.5 mL) and mean mass of cartilage was 5.12 g (± 1.99 g SD, 2.73–9.20 g). Following culture on 25cm^2^ monolayers over 14–21 days, measurements of total DNA using the Picogreen dsDNA assay demonstrated that the concentration of dsDNA was directly proportional to the total quantity of nuclear DNA, and hence the number of cells per flask at harvest (Fig. 4).

We found no statistically significant difference in the quantity of DNA across the different co-culture ratios and monoculture controls. Therefore, it suggests that the level of expression of the genes of interest were dependent on factors other than proliferation rate and cell number.

### Co-culture promotes chondrogenic gene expression

**Fig. 5:**
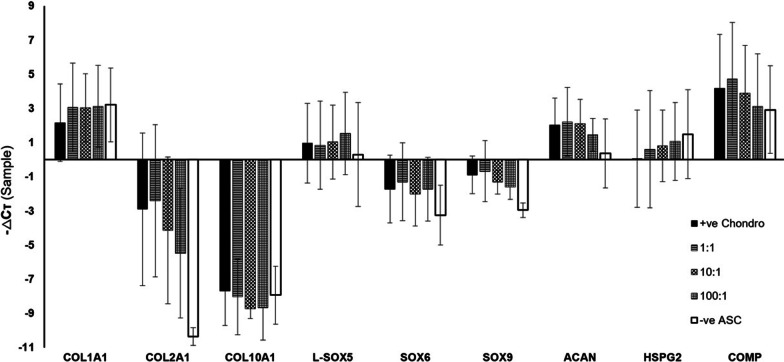
-ΔCτ of different genes of interest showing relative gene expression normalized to HPRT1 (n = 5); positive chondrocyte control (+ ve Chondro), negative ASC control (− ve ASC)

**Fig. 6:**
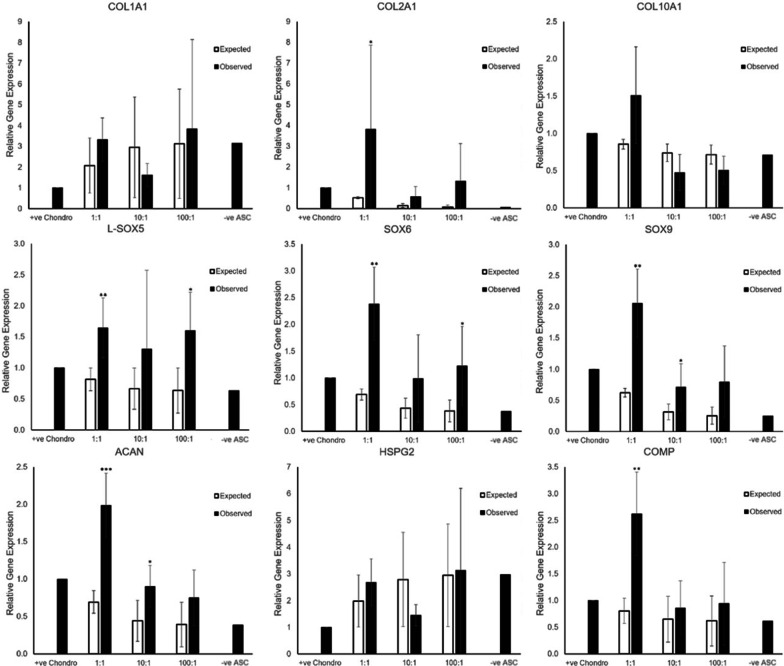
Expected vs Observed levels of expression of chondrogenic genes of interest were different across various co-culture ratios normalized against the positive chondrocyte control (n = 5; Mean ± SD). *p < 0.05, **p < 0.01 comparing between expected and observed gene expression

A chondrogenic panel of three collagen genes (COL1A1, COL2A1 and COL10A1), three SOX genes (L-SOX5, SOX6, SOX9), two proteoglycans, and one extracellular matrix (ECM) protein was designed to study the chondrogenic gene expression of ASC, chondrocyte monocultures and co-cultures at week 2–3. All genes except COL2A1 for ASC monoculture and COL10A1 for any interval are expressed, albeit at various levels of expression for the different genes and cell ratios (Fig. 5). To elucidate whether co-culture has brought about greater than expected chondrogenic gene expression, a novel approach applying pairwise comparison of expected and observed levels of gene expression was performed per gene of interest per co-culture ratio as shown in Fig. 6. Greater expression than expected of genes characteristic of chondrogenic differentiation was seen with co-culture intervals of lower ASC-to-chondrocyte ratios with a statistically significant difference (*p* < 0.05) in the expression of COL2A1 (*p* = 0.004), L-SOX5 (*p* = 0.0061), SOX6 (*p* = 0.0036), SOX9 (*p* = 0.0029), ACAN (*p* = 0.0002), and COMP (*p* = 0.0038) for the 1:1 co-culture ratio. Statistically significant differences of SOX9 (*p* = 0.0412) and ACAN (*p* = 0.0245) only were observed for the 10:1 co-culture ratio, and of L-SOX5 (*p* = 0.0136) and SOX6 (*p* = 0.0426) for the 100:1 ratio. These results suggest that the optimal number of chondrocytes and ASCs for chondrogenic pre-conditioning is near a 1:1 co-culture ratio.

## Discussion

Although the potential of ASCs for chondral and osteochondral repair is well established in vitro and in animal studies, similar efficacy has not been observed in clinical trials. One reason for this may be due to difficulties in culturing ASCs ex vivo to undergo chondrogenic differentiation prior to implantation into chondral lesions. Therefore, new ways of improving the chondrogenic differentiation of ASCs are needed. The use of IFPD ASCs isolated from fat pad for coculture with chondrocytes, with the aim of improving chondrogenic differentiation is novel and has not previously been investigated. Human IFPD ASCs have been shown to engraft with acellular dermal matrix and express chondrogenic genes in vitro [39]. Compared to subcutaneous adipose-derived ASCs, IFPD ASCs exhibit similar proliferative potential, but superior chondrogenic and osteogenic differentiation [40]. IFPD ASCs also appear to have pre-served osteogenic potential with increasing age [41], although the relationship to undergo chondrogenic differentiation with age is less well described. In this study, we have investigated the interaction of chondrocytes and IFPD ASCs with a view to understanding how to optimize the potential of ASCs for improving cartilage repair. Phenotyping of IFPD ASCs by flow cytometry and trilineage differentiation confirmed their multipotent potential while proliferation measured by PicoGreen dsDNA assay ascertained a similar level of proliferation across seeding ratios. We validate that the infrapatellar fat pad represents a feasible and convenient cell-source for the isolation of ASCs for potential therapeutic use.

### Gene expression

In our study, while the IFPD ASC and chondrocyte co-cultures exhibited more variable results than those for monocultures, there was nonetheless evidence demonstrating in-creased chondrogenicity of co-culture than would be expected by ratios derived from monoculture. This is particularly evidenced by the significant increase in expression of COL2A1, L-SOX5, SOX6, SOX9, ACAN, and COMP in a 1:1 IFPD ASC-to-chondrocyte co-culture ratio. The upregulation of these genes is more pronounced in a co-culture ratio of 1:1 IFPD ASC-to-chondrocyte than 10:1 or 100:1. Although it is also seen to a lesser extent in the 10:1 co-culture ratio for SOX9 & ACAN, and in the 100:1 ratio for L-SOX5 & SOX6. This has implications in providing an optimal co-culture ratio near 1:1 when seeding IFPD ASCs to be pre-conditioned by chondrocytes. It may be beneficial for autologous ASCs to be pre-conditioned with high relative ratios of chondrocyte via co-culture, prior to use for the purposes of tissue-engineering and cartilage regeneration.

COMP encodes thrombospondin-5 [42], and contributes to forming the extracellular matrix of cartilage together with type II collagen, aggrecan, and perlecan [43]. L-SOX5, SOX6, and SOX9 encode HMG-box transcription factors that initiate and maintain the commitment of differentiating ASCs on a chondrogenic lineage [44]. The lack of increased expression of COL1A1 and COL10A1 suggests minimal induction of an osteogenic [45] or dedifferentiating phenotype [46] and endochondral ossification respectively.

Therefore, by screening gene expression of collagen, HMG-box transcription factors, and proteoglycan/ECM pertaining to chondrogenic cell fate, this study has provided novel insight into the IFPD ASC and chondrocyte crosstalk and the potential use of pre-conditioned ASCs by autologous co-culture in cellular therapies.

### Possible pathways

Cell-based therapies for cartilage repair centre on the crosstalk between ASCs and en-dogenous chondrocytes. Our data suggests that this may be exploited in vitro to alter the properties of these therapies. Such crosstalk may involve bidirectional signaling influenced by the secretome of both cell types, including cytokines e.g. TGF-β superfamily [47] and exosomes carrying miRNA [48] which are secreted for paracrine induction into their surrounding microenvironment in vivo or into the culture medium in vitro. Although beyond the scope of this study, future media transfer experiments may further elucidate this. Juxtacrine signaling between ASCs and chondrocytes involving cell-to-cell or cell-to-extracellular matrix contact include Notch signaling and Notch-dependent micropatterning facilitated by cell-to-cell contact between both cell types may also play a significant role in chondrogenic differentiation [49, 50], therefore 3D co-culture systems could be a potential way of improving chondrogenesis.

### Limitations

In view of what we know about the gene expression of mature chondrocytes and ASCs, and the relative respective cell ratios used in our experiments, the more likely explanation for the results seen is the effect of chondrocytes on IFPD ASC gene expression. It is however possible that part or all of the results seen may be due to the effect of IFPD ASC on chondrocyte gene expression, therefore, the nature of this interaction remains un-known at present. Furthermore, as we observed no significant difference in chondrogenic gene expression between monocultures, possible de-differentiation of chondrocytes cannot be excluded. Future studies using conditioned media would help better under-stand this paracrine effect. Further assessment of the cellular contents and conditioned media with protein assays may help provide insight into active components of the secretome as well as chondrogenic differentiation. Through cell-sorting techniques, including Fluorescence Activated Cell Sorting (FACS), ASCs and chondrocytes may also be separated following co-culture to allow features such as gene expression and proliferation of each population to be assessed individually. Our experiments nevertheless demonstrate the potential benefit of co-culture on in vitro chondrogenesis.

Different genes are expressed at different levels, and this is a limiting factor for this study. Small increases in gene expression may be overshadowed by the variance of results. This is limited by the power of the study since observed power is inversely proportional to the p-value due to the small sample size of n = 5. In particular, significant variance was ob-served in the results of the proliferation assay, suggesting that greater sample sizes are required to reliably discern any true difference.

In vivo cell phenotype of chondrocytes and in situ IFPD ASCs may be affected by the chronic stress and inflammatory effects of osteoarthritis, leading to reduced proliferative capacity. Indeed, the fat pad has been implicated as a disease-relevant tissue in the pathogenesis of osteoarthritis, possibly contributing to global joint inflammation and sensitization of the joint. In spite of this, human IFPD ASCs isolated from osteoarthritic joints have been shown to exert chondrogenic capacity similar to that of non-osteoarthritic joints [51]. When exposed to pro-inflammatory cytokines, IFPD ASCs adopt an im-munomodulatory phenotype, and are able to reduce joint inflammatory in vivo [52]. However, it is important to note that whilst ASCs pre-conditioned with inflammatory cytokines can display enhanced immunomodulatory effects, their proliferative capacity may be reduced [53]. The secretome and inherent chondrogenicity of chondrocytes harvested from the osteoarthritic joint may differ from those derived from non-osteoarthritic sites. This could be investigated through an IFPD ASCs co-culture with chondrocytes derived from joints affected with osteoarthritis compared with those originating from unaffected joints.

In vitro conditions may not necessarily be consistent with the in vivo microenvironment as articular cartilage is avascular and hypoxic [54]. Therefore, future in vitro chondrogenic co-culture experiments that closely mimic the in vivo hypoxic intraarticular environment should be considered [55].

## Conclusions

Our study shows that pre-conditioning IFPD ASCs in co-culture with chondrocytes can enhance chondrogenic gene expression in vitro, and this effect is greater when seeding at a lower IFPD ASC-to-chondrocyte ratio. The observed differences in gene expression did not appear to be due to differences in cell number and proliferation rate, therefore we speculate that paracrine effects and juxtacrine signaling pathways between ASCs and chondrocytes are likely to be involved. Our findings suggest a need to investigate alternative culture conditions for ASCs in clinical trials of intraarticular therapy, potentially through co-culture with chondrocytes or incubation in chondrocyte-conditioned media prior to injection of the ASCs into the target site. There is thus a need to further demonstrate the efficacy of pre-conditioned ASCs by co-culture in pre-clinical, animal, and clinical trials.

## Supplementary Information

**Additional file 1: Table S1.** Fluorophores used for characterisation of cell-surface markers of ASCs. **Table S2.** Composition of Chondrogenic media. **Table S3.**Composition of Osteogenic media. N.B. For adipogenic differentiation, 1mL of the adipogenic medium from the differentiation kit was applied to each well. **Table S4.** Flow cytometry antibody combinations and gating percentages

## Data Availability

The datasets used and/or analysed during the current study are available from the corresponding author on reasonable request.

## Abbreviations

△Cτ: Cycle threshold
DMEM: Dulbecco’s modified eagle medium
ECM: Extracellular matrix
FACS: Fluorescence activated cell sorting
FBS: Fetal bovine serum
IFPD: Infrapatellar fat pad-derived
MSC: Mesenchymal stromal cells
p0: Passage 0
