# Supplementary material for: Infrapatellar fat pad adipose-derived stem cells co-cultured with articular chondrocytes from osteoarthritis patients exhibit increased chondrogenic gene expression
Source: Cell Commun Signal. 2022 Feb 12;20:17. doi: 10.1186/s12964-021-00815-x (PMC8841120; doi:10.1186/s12964-021-00815-x)
Supplement: Supplementary file 2 — Additional file 1: Table S1. Fluorophores used for characterisation of cell-surface markers of ASCs. Table S2. Composition of Chondrogenic media. Table S3.Composition of Osteogenic media. N.B. For adipogenic differentiation, 1mL of the adipogenic medium from the differentiation kit was applied to each well. Table S4. Flow cytometry antibody combinations and gating percentages [file 12964_2021_815_MOESM2_ESM.docx]

**Additional file:**

**Table S1. Fluorophores used for characterisation of cell-surface markers of ASCs**

| For use in BD Fortessa Flow Cytometer | | Surface Marker | Fluorophore | ul required per 100ul staining volume |
| --- | --- | --- | --- | --- |
| 488  Blue emission | 530/30 | HLA-DR | FIT-C | 0.5 |
|  | 695/40 | CD73 | PerCP-eFluor710 | 1.0 |
| 561  Yellow emission | 670/14 | CD19 | PE | 1.0 |
| 405 Violet emission | 525/50 | Non-viable Cells | Zombie Aqua | 0.5 |
|  | 610/20 | CD34 | BV605 | 1.0 |
|  | 660/20 | CD105 | BV650 | 0.5 |
|  | 780/60 | CD45 | BV785 | 1.0 |
| 640 Red emission | 660/20 | CD14 | APC | 1.0 |
|  | 730/45 | CD90 | AlexaFluor700 | 1.0 |

**Table S2. Composition of Chondrogenic media**

| Chondrogenic | Solution | Volume or Volume ratio | Volume for 10mL stock per media change |
| --- | --- | --- | --- |
| Stock Solution  3-month shell life | High Glucose DMEM (4.5g/mL) w/ Glutamax | 500mL | 10mL |
|  | P/S | 5mL |  |
|  | Sodium Pyruvate | 5mL |  |
|  | ITS (insulin) | 5mL |  |
|  | Proline (40 ug/mL) | 1mL |  |
| Prepared fresh per media change | TGFβ1 (2-week shell life) | 1:200 | 50µL |
|  | Vitamin C (1-day shell life) | 1:500 | 20µL |
|  | Dexamethasone | 1:10000 | 1µL |

**Table S3. Composition of Osteogenic media**

| Osteogenic | Solution | Volume or Volume ratio | Volume for 10mL stock per media change |
| --- | --- | --- | --- |
| Stock Solution  3-month shell life | High Glucose DMEM (4.5g/mL) w/ Glutamax | 500mL | 9mL |
|  | FCS | 50mL |  |
|  | P/S | 5mL |  |
| Prepared fresh per media change | BGP | 1:9 | 1mL |
|  | Vitamin C (1-day shell life) | 1:90 | 111µL |
|  | Dexamethasone | 1:9000 | 1.1µL |

N.B. For adipogenic differentiation, 1mL of the adipogenic medium from the differentiation kit was applied to each well.

Table S4. Flow cytometry antibody combinations and gating percentages.

| Gate | Number | Percentage Gated |
| --- | --- | --- |
| ALL | 183985 | 100.00 |
| FSC-A | 18122 | 11.05 |
| FSC-W | 16737 | 92.53 |
| Zombie Aqua | 13922 | 83.44 |
| CD14-/CD19- | 13541 | 97.13 |
| CD34-/CD45- | 11470 | 82.11 |
| CD34+/CD45- | 2208 | 16.11 |
| CD73+/CD90+ | 12928 | 92.45 |
| CD105+/HLA-DR- | 13216 | 94.90 |
